# Effect of oligonol, a lychee‐derived polyphenol, on skeletal muscle in ovariectomized rats by regulating body composition, protein turnover, and mitochondrial quality signaling

**DOI:** 10.1002/fsn3.2750

**Published:** 2022-03-11

**Authors:** Jeong Hun Kim, Hyangkyu Lee, Ji Min Kim, Byung‐Joo Lee, In‐Joo Kim, Kyoungjune Pak, Yun Kyung Jeon, Keunyoung Kim

**Affiliations:** ^1^ 220312 Biomedical Research Institute Pusan National University Hospital Busan Korea; ^2^ Biobehavioral Research Centre Mo‐Im Kim Nursing Research Institute College of Nursing Yonsei University Seoul Korea; ^3^ Pusan National University Medical Research Institute Pusan National University School of Medicine Pusan National University Yangsan Korea; ^4^ Department of Otorhinolaryngology‐Head and Neck Surgery Pusan National University School of Medicine Pusan National University Busan Korea; ^5^ 220312 Department of Nuclear Medicine and Biomedical Research Institute Pusan National University Hospital Busan Korea; ^6^ 220312 Department of Internal Medicine and Biomedical Research Institute Pusan National University Hospital Busan Korea

**Keywords:** body composition, mitochondrial dynamics, ovariectomy, polyphenols, skeletal muscle

## Abstract

Oligonol is a low‐molecular‐weight polyphenol product derived from lychee (*Litchi chinensis* Sonn.) fruits. This study was focused on the effects of oligonol on the skeletal muscle of ovariectomized rats. We randomly divided female Sprague–Dawley rats into three groups: a sham surgery control group (Sham), an ovariectomy (OVX) group, and an OVX group treated with oligonol (OVX + Oligonol). Oligonol was intraperitoneally administrated at 30 mg/kg daily for 6 weeks. Oligonol treatment after OVX decreased body weight and fat mass, regulated lipid metabolism in skeletal muscle, without loss of lean mass and bone. Bone turnover was not affected by oligonol. In protein synthesis and degradation, oligonol increased the levels of the mammalian target of rapamycin and its downstream targets, eukaryotic initiation factor 4E‐binding protein 1 and 70‐kDa ribosomal protein S6 kinase, and it stimulated the expression of ubiquitin‐proteasome pathway proteins, the forkhead box transcription factors of the class O and the muscle ring‐finger protein‐1. Moreover, oligonol treatment enhanced mitochondrial biogenesis and dynamics. Thus, our results indicated that oligonol treatment had beneficial effects on the skeletal muscle in an estrogen‐deficiency rat model.

## Graphical abstract

Oligonol is a low‐molecular‐weight polyphenol product derived from lychee (*Litchi chinensis* Sonn.) fruits. It empirically demonstrates the efficacy of the natural plant product oligonol in the amelioration of sarcopenic obesity associated with estradiol deficiency resulting from ovariectomy by regulating body composition, protein turnover, and mitochondrial quality signaling.

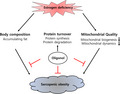

## 1 INTRODUCTION

Sarcopenia, an age‐related decrease in muscle mass and function, often co‐occurs with other diseases and is one of the most important health problems in older people (Cruz‐Jentoft et al., 2019). Sarcopenia was originally identified as a muscle mass loss, but its definition has been recently changed to consider all factors related to muscle mass, quality, and strength (Cruz‐Jentoft et al., 2019). Indeed, sarcopenia occurs predominantly in older people, but a life course model of sarcopenia additionally includes the determinants of maximal muscle mass and strength in adulthood as contributing factors to sarcopenia (Sayer et al., 2008).

Generally, women have a higher risk of developing sarcopenia than men due to their lower maximal muscle mass and weaker muscle strength (Dennison et al., 2017; Dodds et al., 2014). Muscle strength is significantly associated with osteoporosis, fractures, and falls in postmenopausal women (Sjoblom et al., 2013). The menopausal status is also associated with a significant decrease in bone function, along with a decrease in muscle mass (Juppi et al., 2020; Messier et al., 2011; Sipila et al., 2020). Estrogen deficiency leads to the sarcopenic obesity phenotype by accumulating fat (Yoon et al., 2020). Long‐term estrogen deficiency is accompanied by muscle atrophy with changes in the distribution of myofiber types (Kitajima & Ono, 2016). Sarcopenic obesity reduces muscle performance in middle‐aged women and is a risk factor for increased falls in postmenopausal women (Follis et al., 2018; Moreira et al., 2016).

Nutritional supplementation is performed as an intervention to prevent sarcopenia, and especially polyphenols with anti‐inflammatory and antioxidant properties are suggested as potential candidate compounds for sarcopenia treatment by improving mitochondrial function (Salucci & Falcieri, 2020). Oligonol derived from lychee (*Litchi chinensis* Sonn.) fruit extracts is an enriched polyphenol product that can reduce ROS‐related inflammation and lipid metabolism (Noh et al., 2011; Park et al., 2015). Oligonol can protect against organ damage, especially in the salivary glands, pancreas, liver, and skeletal muscle (Kim et al., 2021; Lee et al., 2021; Liu et al., 2017; Park et al., 2018). Recently, oligonol has been shown to control proteolysis and enhance skeletal muscle quality via pathways related to mitochondrial dynamics (Chang et al., 2019; Liu et al., 2017). However, the effect of polyphenol administration on body composition has been rarely studied. In this study, we investigated whether administering oligonol affects the quantity and quality of skeletal muscle. We specifically studied the effect of oligonol in an ovariectomized rat model that included rats subjected to ovariectomy (OVX) as the OVX group, along with ovariectomized rats receiving oligonol after surgery as the OVX + Oligonol group, and control rats that underwent sham surgery as the Sham group.

## 2 METHODS

### 2.1 Animals

Twenty‐four female Sprague–Dawley rats (Koatech, Gyeonggi‐do, Korea), 9 weeks old, were used. Specifically, after 1 week of acclimatization, rats were randomly assigned to three groups: Sham group, rats that underwent sham surgery (*n* = 8); OVX group, rats that received OVX, (*n* = 8); OVX + oligonol group, rats receiving OVX and treated with oligonol (*n* = 8). Animals were euthanized using CO_2_ gas 6 weeks after surgery. All rats were maintained under a light/dark cycle of 12 h with ad libitum access to rat chow and water.

### 2.2 Ethics statement

All animal procedures were performed following relevant guidelines and regulations. The animal care and research protocols were based on the principles and guidelines adopted by the Guide for the Care and Use of Laboratory Animals (National Institutes of Health publication), and all animal experiments were documented following the ARRIVE reporting guidelines. The study was approved by the Institutional Animal Care and Ethics Committee of Pusan National University Hospital (PNUH‐2020‐172).

### 2.3 Establishment of the ovariectomized rat model

Prior to performing operation, rats were injected with tramadol 5 mg/kg intraperitoneally under isoflurane inhalation anesthesia. Then, an approximately 1 cm incision was made along the midline of the abdomen to expose both ovaries. In the OVX group, both ovaries were resected and the abdominal cavity was closed. In the Sham group, sham surgery was performed to expose the ovaries and close the abdominal cavity without excision. In the OVX + Oligonol group, oligonol (KCF Korea Co.) diluted with phosphate‐buffered saline was intraperitoneally injected into ovariectomized rats at 30 mg/kg daily for 6 weeks after surgery.

### 2.4 Body composition

The rat body composition was scanned using a dual‐energy X‐ray Absorptiometry (DXA) system (InSight; Osteosys R&D Center) under anesthesia with an isoflurane inhalation vaporizer (Matrix VIP 3000™, Midmark Co.). For the whole‐body composition analysis, the supplied software generated the body weight (g), bone mineral density (g/cm**^2^**), fat mass (g), fat proportion (%), and lean mass (g) of regions of interest.

### 2.5 Enzyme‐linked immunosorbent assay (ELISA)

Upon euthanasia, whole blood samples were obtained by cardiac puncture and processed by centrifugation at 400 *g* for 15 min at 4°C. Serum fractions were collected and stored at −20°C until analysis. Serum concentrations of osteocalcin and C‐telopeptide of collagen Type 1 (CTX‐1) were measured using ELISA kits (MyBioSource). The absorbance was read at 450 nm, following the manufacturer's instructions.

### 2.6 Histology and morphometric analysis

The soleus muscle tissue was isolated from each rat and treated by fixing overnight in 10% neutral‐buffered formalin. Paraffin embedding (Leica, TP1020, semi‐enclosed benchtop tissue processor) and dispensing (Leica, EG1150H, heated paraffin‐embedded module) was performed using an automatic tissue processor. For staining analysis, slides were deparaffinized with xylene and then hydrated through a series of washes with sequential ethanol and water. The cut sections were stained with hematoxylin and eosin (H&E) and Sirius red (Abcam; catalog number #ab150681). For quantitative analysis of myofiber size, 50 myofiber cross‐sections per picture were evaluated and the mean of seven randomly selected non‐overlapping areas was statistically analyzed using ImageJ software.

### 2.7 Electron microscopy

The skeletal muscle tissue was pre‐fixed with 2.5% glutaraldehyde (4°C, phosphate buffer, pH 7.4) and post‐fixed with 1% osmium tetroxide in the same buffer. The fixed material was subjected to a series of dehydration processes with ethyl alcohol and embedded in an epoxy resin (Epon 812 mixture). To select the observation site, a thick section (1 μm) was stained with 1% toluidine blue and confirmed through an optical microscope. Thin sections (50–60 nm) were prepared using an ultramicrotome (EM UC7, Leica), double stained with uranyl acetate and lead citrate, and examined with a transmission electron microscope (JEM‐1200EXII, JEOL).

### 2.8 Western blot analysis

Whole soleus muscle tissue lysates were protein‐extracted using a lysis buffer containing protease and phosphatase inhibitors (Translab). Protein content was quantified using the BCA assay (Thermo Fisher Scientific), and each sample at the same concentration was denatured in Laemmli's sample buffer at 100°C for 5 min. Each sample (30 µg) was loaded on 10% SDS‐PAGE (Elpis Bio) and transferred to a polyvinylidene fluoride (PVDF) membrane. Transferred membranes were blocked using 5% skim milk for 1 h at room temperature and then incubated with primary antibody overnight at 4°C. The primary antibody was then thoroughly washed with TBS‐T and incubated with the secondary antibody for 1 h at room temperature. Antibodies used are listed in Table 1. To visualize the immunoblot signal, it was developed on an ATTO Chemiluminescence Imaging Device (AE‐9150 Ez‐capture II, Tokyo, Japan) using Amersham ECL Select (GE Healthcare). GAPDH protein levels were normalized to the loading control. Raw data were quantified using the manufacturer's analysis tool (Image Analyzer CS4).

**TABLE 1:** List of antibodies

	Host	Source (Cat No.)	Dilution
SREBP‐1c (Sterol regulatory element‐binding protein‐1)	Mouse	Santa cruz (sc‐365513)	1:400
CPT‐1α (Carnitine palmitoyltransferase 1α)	Rabbit	Abclonal (A5307)	1:1,000
mTOR (Mammalian target of rapamycin)	Rabbit	Cell signaling (#2983)	1:1,000
phospho‐mTOR (Phosphorylated mammalian target of rapamycin)	Rabbit	Cell signaling (#5536)	1:1,000
phospho‐70S6 kinase (Phosphorylated ribosomal protein 70 kDa S6 kinase 1)	Rabbit	Cell signaling (#9234)	1:1,000
phospho‐4EBP1 (Phosphorylated eukaryotic translation initiation factor 4E‐binding protein 1)	Rabbit	Cell signaling (#2855)	1:1,000
MuRF‐1 (Muscle RING‐finger protein‐1)	Rabbit	ECM bioscience (MP3401)	1:1,000
PGC‐1α (Peroxisome proliferator‐activated receptor gamma coactivator 1‐alpha)	Rabbit	Abcam (ab54481)	1:2,000
NRF2 (Nuclear factor erythroid 2‐related factor 2)	Rabbit	Abclonal (A1244)	1:1,000
OPA1 (Optic atrophy 1)	Rabbit	Abclonal (A9833)	1:1,000
MFN2 (Mitofusin 2)	Rabbit	Abclonal (A12771)	1:1,000
DRP1 (Dynamin‐related protein 1)	Rabbit	Abclonal (A2586)	1:1,000
FOXO (Forkhead box transcription factors of the class O)	Rabbit	Abclonal (A2934)	1:1,000
Cytochrome C	Rabbit	Abclonal (A0225)	1:1,000
VDAC1 (Voltage‐dependent anion channel protein 1)	Rabbit	Abclonal (A19707)	1:1,000
GAPDH (Glyceraldehyde 3‐phosphate dehydrogenase)	Mouse	Santa cruz (sc‐365062)	1:2,000
Anti‐mouse IgG HRP	Rabbit	Santa cruz (sc‐516102)	1:4,000
Anti‐rabbit IgG HRP	Goat	Cell signaling (#7074)	1:2,000

### 2.9 Statistical analysis

Statistical analysis was performed with GraphPad Prism 7.0. All quantitative data are presented as mean ± standard error. Shapiro–Wilk normality test and Levene's test were used to verify that the data distribution has normality and homogeneity of variance. One‐way analysis of variance (one‐way ANOVA) was used to determine significant differences between groups, followed by Bonferroni's post hoc analysis. *p* < .05 was considered statistically significant.

## 3 RESULTS

### 3.1 Effects of oligonol on body composition and lipid metabolism

**FIGURE 1:**
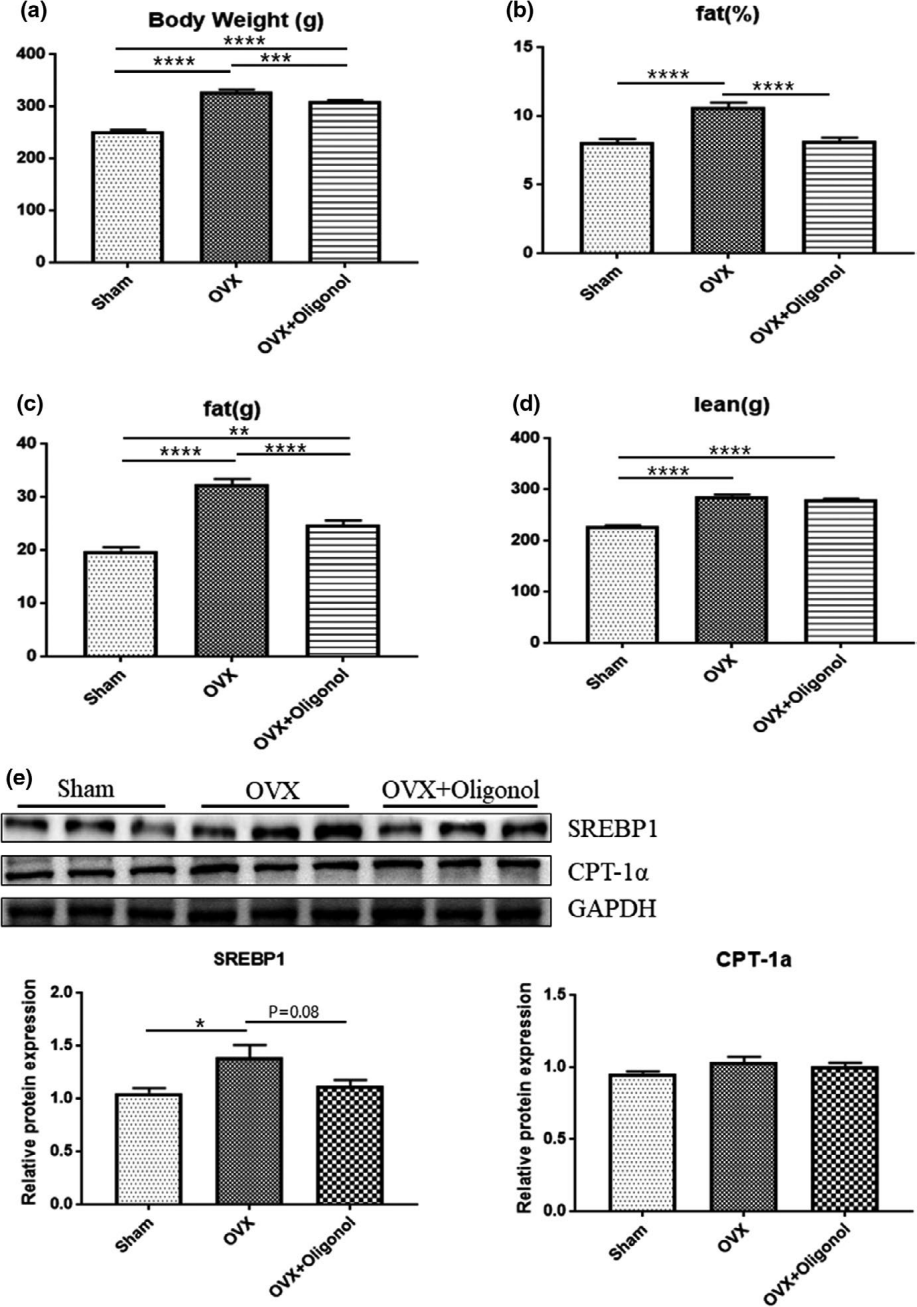
Body composition and lipid metabolism. The body composition analysis included (a) body weight, (b) fat proportion (c), fat mass (d), and lean mass; (e) representative immunoblotting image and morphometric analysis of SREBP1 and CPT‐1α. Protein expression data were normalized to the GAPDH expression levels; *p < .05, **p < .01, ***p < .001, and ****p < .0001

We investigated the effect of oligonol on the body composition of ovariectomized rats using DXA, as shown in Figure 1. We have previously reported that OVX caused an increase in fat mass, muscle mass, and whole‐body fat proportion (Yoon et al., 2020). Based on the DXA analysis in this study, the body weight was significantly lower in the OVX + Oligonol group than in the OVX group, and, moreover, the body fat mass did not significantly differ between the OVX + Oligonol and the Sham group. The OVX group had a loss in lean mass that we did not observe in the OVX + Oligonol group. We further used immunoblotting to assess the expression of lipid metabolism regulators in the skeletal muscle using the SREBP1 protein as lipogenesis marker and the CPT‐1α protein as fatty acid oxidation marker. The SREBP1 expression level was significantly elevated in the OVX group (*p* < .05), whereas oligonol treatment tended to decrease its expression level (*p* = .08) compared with that in the Sham group. The CPT‐1α expression level was similar among all study groups.

### 3.2 Effects of oligonol on bone mineral density and bone metabolism

**FIGURE 2:**
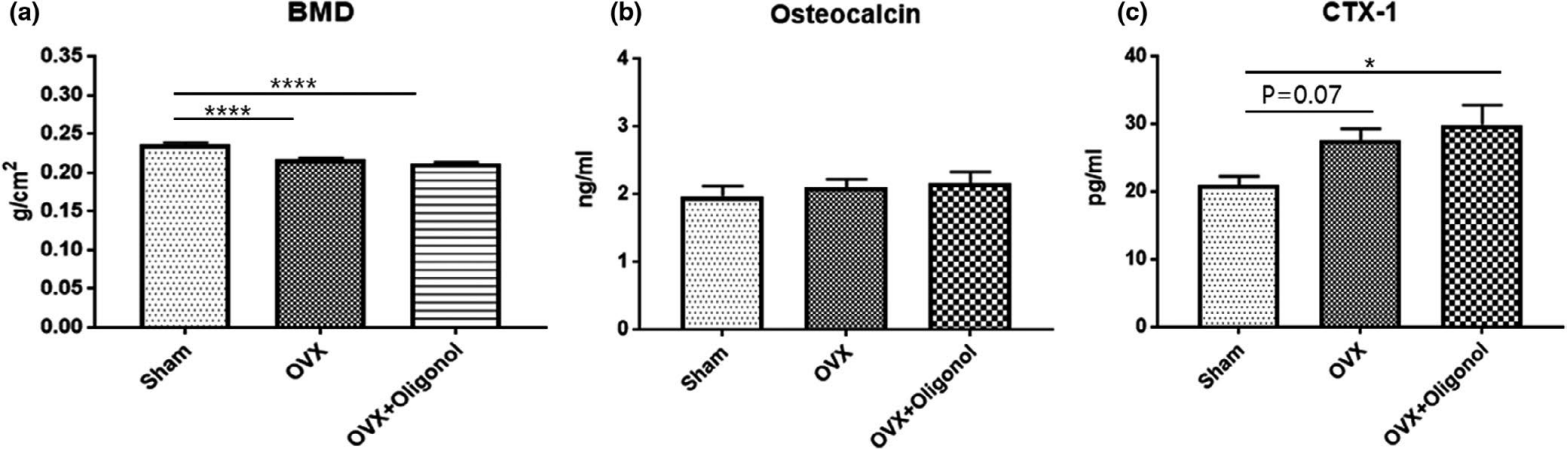
Bone mineral density and bone turnover. (a) Bone mineral density, (b) serum osteocalcin, and (c) serum CTX‐1; *p < .05

Next, we used DXA to examine bone mineral density, and we assessed bone turnover by performing an ELISA analysis to measure the serum levels of osteocalcin and CTX‐1 as markers for bone formation and resorption, respectively (Figure 2). As expected, we found that the bone mineral density was lower in the OVX group than in the Sham group (*p* < .001), but we did not observe any significant difference in bone density between the OVX + Oligonol group and the OVX group (*p* > .05). The serum osteocalcin level was similar among all groups (*p* > .05). However, there was an elevated serum CTX‐1 level in both the OVX (*p* = .07) and the OVX + Oligonol group (*p* < .05), compared with that in the Sham group, but it did not differ between the two ovariectomized groups (*p* > .05).

### 3.3 Effects of oligonol on microscopic findings

**FIGURE 3:**
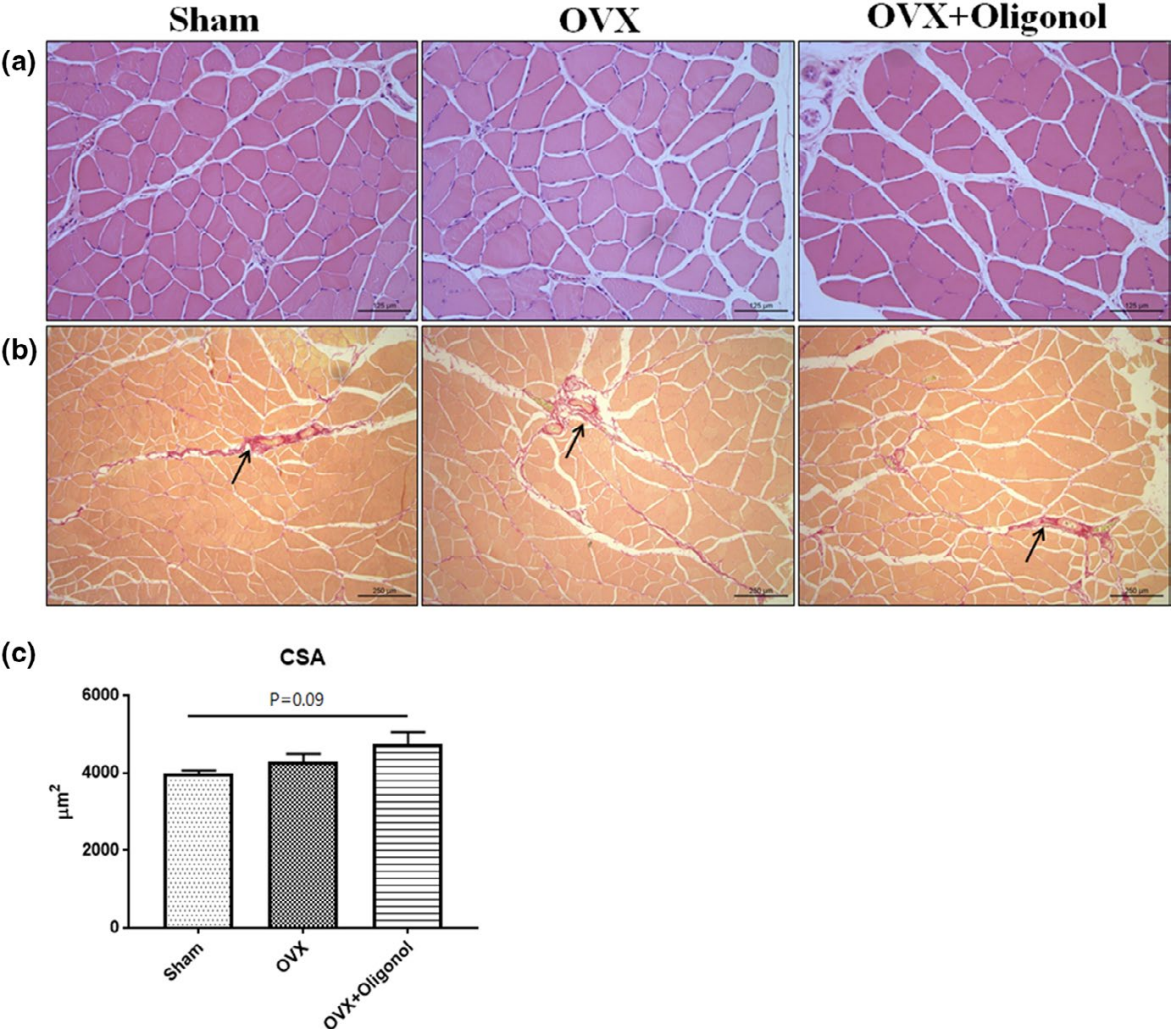
Histological findings. (a) Hematoxylin and eosin (H&E) staining, (b) Sirius red staining (black arrow), and (c) cross‐sectional area in the soleus muscles (*n* = 7 per group). Scale bars = 125 μm; magnifications, 20× for H&E staining and 10× for Sirius red staining

The skeletal muscle fiber is typically surrounded by an extracellular matrix (Csapo et al., 2020). Here, we assessed the skeletal muscle morphology by identifying extracellular matrix components using H&E staining and Sirius red staining (Figure 3a,b). We observed that the area stained by Sirius red was similar among the groups. The cross‐sectional area (CSA) was measured to detect a size change in the myofiber (Figure 3c). The OVX + Oligonol group tended to have the highest CSA, but there was no statistical significance.

### 3.4 Effect of oligonol on protein turnover

**FIGURE 4:**
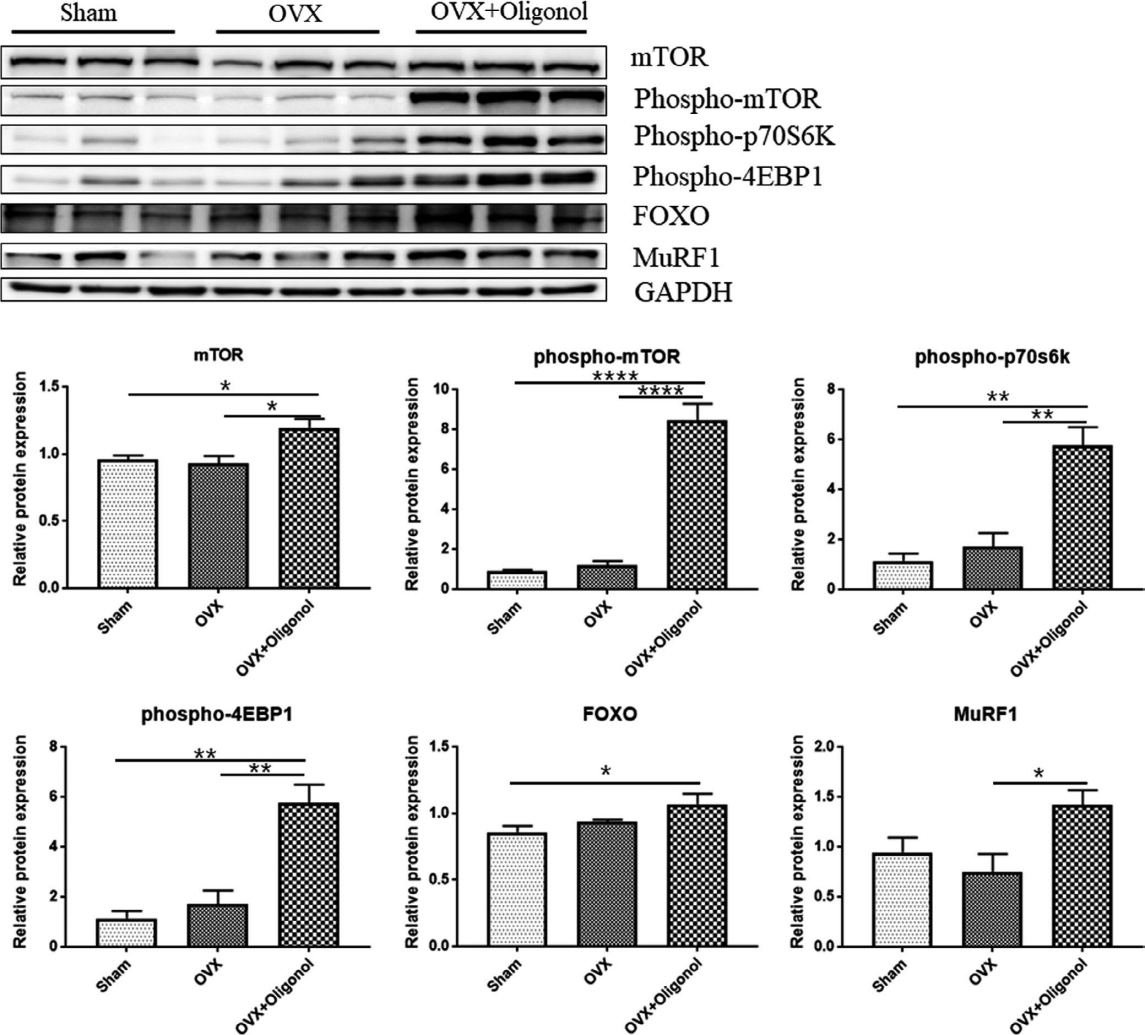
Protein turnover. (a) Representative images of immunoblotting for soleus muscle, (b) mTOR protein expression, (c) phospho‐mTOR protein expression, (d) phospho‐p70s6k protein expression, (e) phospho‐4EBP1 protein expression, and (f) MuRF1 protein expression. Protein expression data were normalized to the GAPDH expression levels; *p < .05, **p < .01, and ***p < .001

We used immunoblotting to evaluate whether the oligonol treatment affected the protein turnover (protein synthesis and degradation) (Figure 4). Protein turnover is regulated by signaling via the mammalian target of rapamycin (mTOR) and the ubiquitin‐proteasome pathway, which is responsible for protein degradation (Collins et al., 2019; Park et al., 2016). Among the three groups, the OVX + Oligonol group had the most elevated protein levels of mTOR (*p* < .05) and phospho‐mTOR (*p* < .0001) with statistical significance. The expression levels of downstream mTOR targets involved in protein synthesis initiation, the phosphorylated ribosomal protein 70 kDa S6 kinase 1 (phospho‐p70s6 kinase) and the phosphorylated eukaryotic translation initiation factor 4E‐binding protein 1 (phospho‐4EBP1), were higher in the OVX group than in the Sham group, but they were even significantly elevated in the OVX + Oligonol group (*p* < .01). Protein levels of the forkhead box transcription factors of the class O (FOXO) and the muscle ring‐finger protein‐1 (MuRF1), which indicates ubiquitin‐proteasome‐mediated protein degradation, did not significantly vary between the OVX and the Sham group but tended to be elevated in the OVX + Oligonol group (*p* = .07).

### 3.5 Effect of oligonol on ultrastructure

**FIGURE 5:**
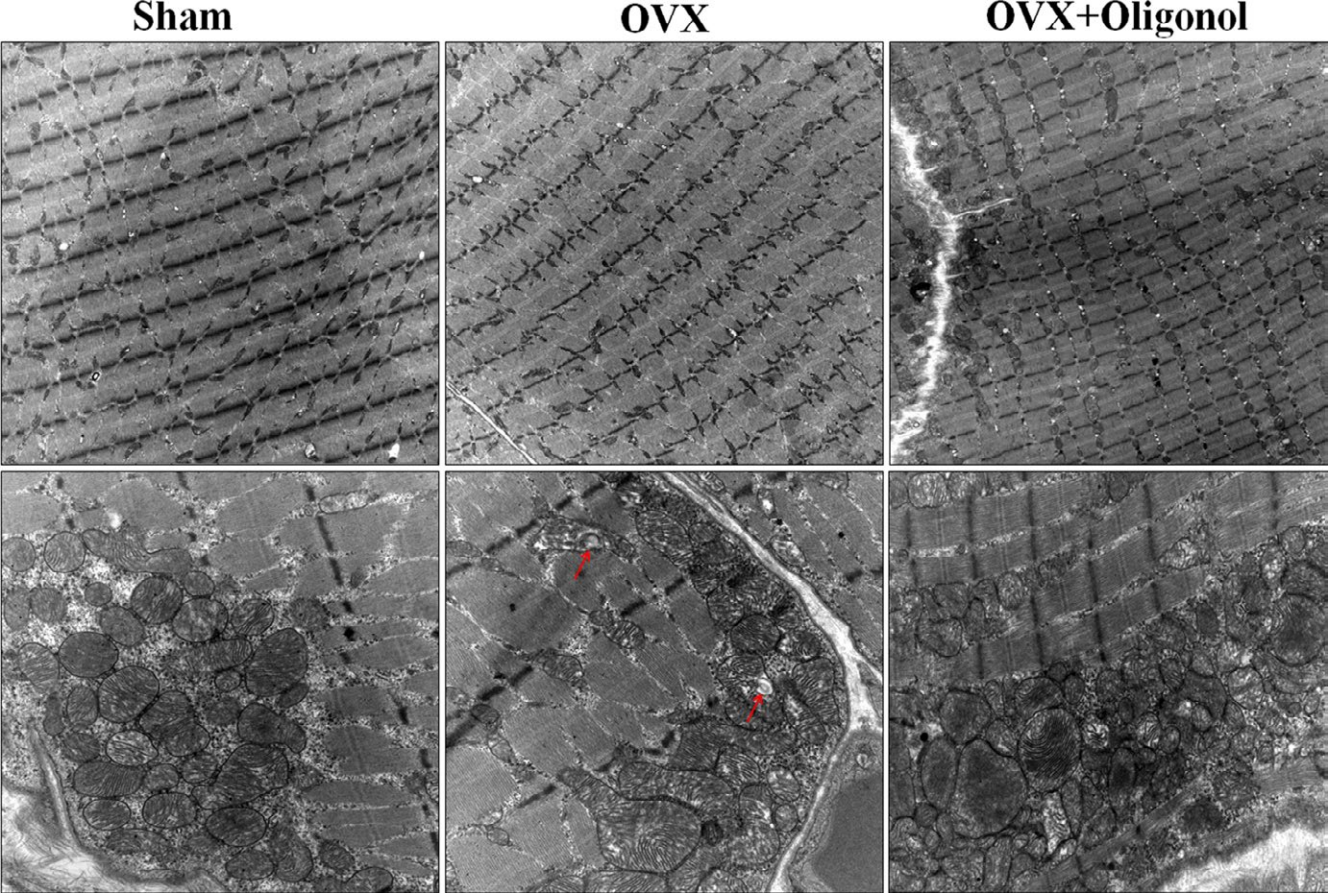
Electron microscopy. Representative electron micrograph of the subsarcolemmal and intermyofibrillar area of the soleus muscle. OVX was disorganized the inner mitochondrial membrane (red arrow), and oligonol supplementation (OVX + Oligonol) was increased the size of mitochondria.; magnification, 20,000×

We next assessed the skeletal muscle ultrastructure by transmission electron microscopy (Figure 5). There were no detectable differences in the myofibril structure among the groups. We observed disintegrated cristae in the OVX group, and the OVX + Oligonol group had an increased number of mitochondria.

### 3.6 Effect of oligonol on mitochondrial biogenesis and dynamics

**FIGURE 6:**
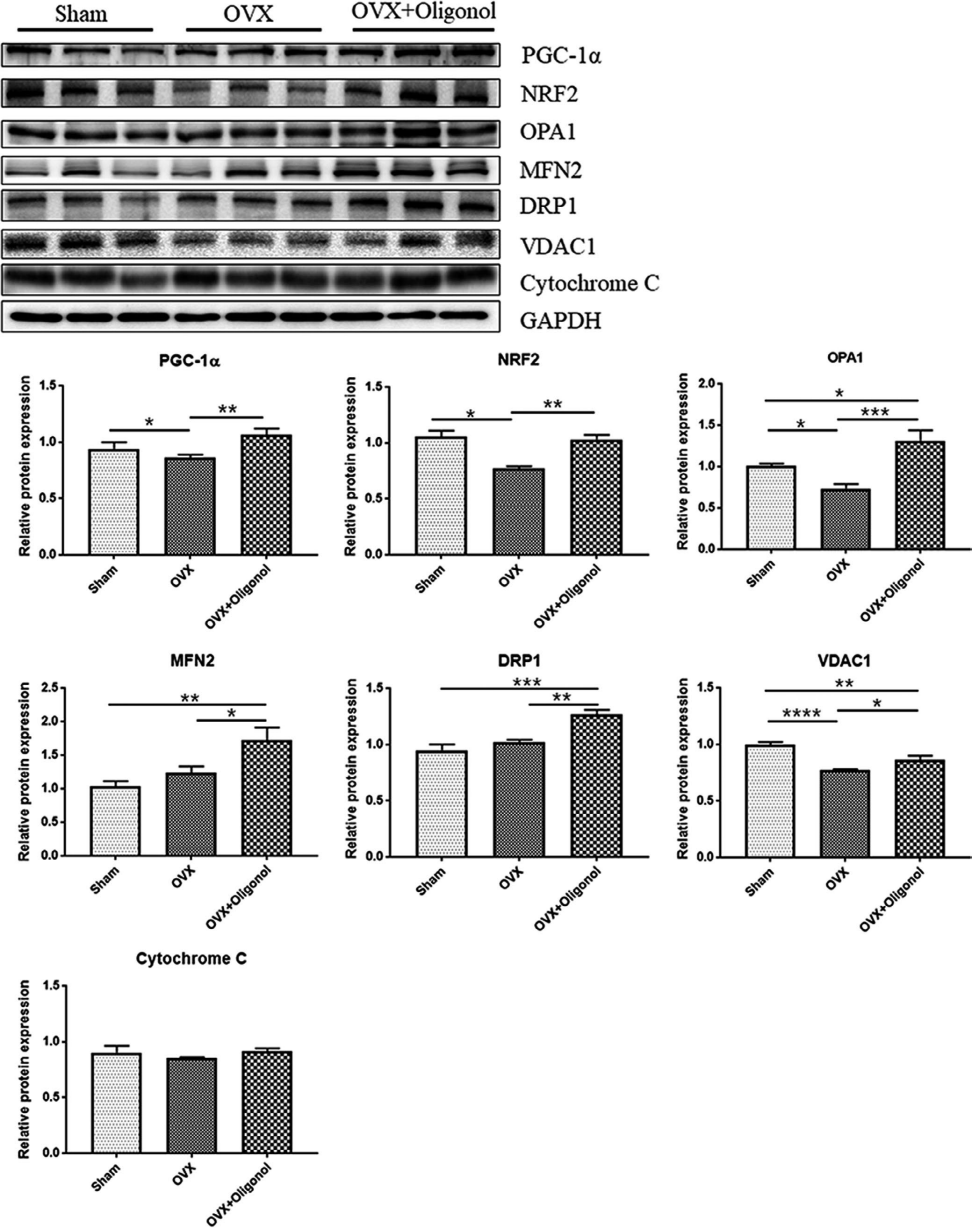
Mitochondrial biogenesis and dynamics. Representative immunoblotting image and morphometric analysis of mitochondrial biogenesis and dynamics in the soleus muscle. Protein expression data were normalized to the GAPDH expression levels; *p < .05, **p < .01, and ***p < .001

To further evaluate the effect of oligonol on mitochondrial quality control, we performed an immunoblotting analysis (Figure 6). Peroxisome proliferator‐activated receptor gamma coactivator 1‐alpha (PGC‐1α) and nuclear factor erythroid 2‐related factor 2 (NRF2) levels were significantly lower in the OVX group (*p* < .05) than in the Sham group, but oligonol treatment (OVX + Oligonol group) restored the PGC‐1α and NRF2 levels to those in the Sham group (*p* < .001). The optic atrophy 1 (OPA1) level was significantly lower in the OVX group than in the Sham group (*p* < .05), whereas it was increased by oligonol treatment (OVX + Oligonol group) (*p* < .001). The level of mitofusin 2 (MFN2) was similar in the OVX and the Sham group, whereas that in the OVX + Oligonol group was the highest among the groups. The dynamin‐related protein 1 (DRP1) level was similar in the Sham and the OVX group, but it was significantly increased in the OVX + Oligonol group. Moreover, the VDAC1 expression level was significantly lower in the OVX group than in the Sham group but was significantly increased by the oligonol treatment. The expression of cytochrome c was similar among all groups.

## 4 DISCUSSION

Sarcopenia has been recently recognized as a serious disease that not only threatens the quality of life but also determines mortality. Estrogen is a key factor in the overall maintenance of the number of satellite cells and muscle function, and its insufficiency affects muscle strength and regeneration (Collins et al., 2019; Ikeda et al., 2019; Kitajima & Ono, 2016). There is evidence that estrogen plays a protective role in the skeletal muscle against apoptosis triggered by mitochondrial signaling (Collins et al., 2019). Our study revealed molecular mechanisms mediating the effects of oligonol supplementation on body composition, protein turnover, and mitochondrial quality in a postmenopausal rat model.

We initially evaluated body composition to identify changes in muscle mass. As in our previous study (Yoon et al., 2020), ovariectomy did not cause a change in the lean mass proportion at extremities and trunk sites, but there was an increase in fat mass percentage, along with an increase in lean mass. The body weight‐adjusted lean mass index decreased after ovariectomy. However, body composition was affected by the oligonol treatment. Remarkably, substantial changes in body weight and the accumulated fat occurred without muscle loss under oligonol treatment. An increase in lipogenesis due to OVX was indicated by the elevated expression of SREBP1; however, under oligonol treatment, there was a tendency to reduce the SREBP1 level. Several other studies have reported that oligonol regulates lipid metabolism and reactive oxygen species (ROS)‐related oxidative stress (Noh et al., 2011; Park et al., 2015, 2016). A recent study demonstrated that the administration of resveratrol suppressed the increase in body weight and fat mass induced by a high‐fat diet (Niu et al., 2021). In a clinical trial, administration of polyphenols extracted from *Ecklonia cava* reduced fat mass and regulated lipid metabolism in subjects with abdominal obesity (Lee et al., 2018). Similarly, oligonol supplementation has been shown to control body weight and improve the blood lipid profile in obese women (Bahijri et al., 2018). Specifically, oligonol inhibited intramuscular lipid accumulation by increasing the AMPKα activity and PPARα expression (Liu et al., 2017). Taken together, these findings indicated that oligonol reduced the risk of sarcopenic obesity by inhibiting lipid accumulation without muscle loss. Current evidence indicates that weight loss is associated with a decrease in bone density (Hunter et al., 2014). However, although oligonol treatment decreased the body weight, it did not affect the bone mineral density and bone turnover after ovariectomy. Antioxidant compounds are expected to play an important role in the management of cellular stress due to the detrimental effects induced by ROS in bone diseases (Agidigbi & Kim, 2019). However, the effects of polyphenols on bone density and bone turnover are not well understood (Austermann et al., 2019). Nevertheless, the fact that the bone loss caused by weight‐bearing restriction did not increase under oligonol treatment suggested a partial inhibition of bone resorption.

Muscle mass‐determining factors are typically involved in regulating the balance of protein synthesis and degradation. In this study, there was no significant difference in myofiber size between the Sham and the OVX group. Furthermore, we found that the mTOR and phospho‐mTOR levels did not differ between the Sham and the OVX group, whereas phospho‐p70S6k and phospho‐4EBP1 expression tended to be upregulated in the OVX group. Although oligonol increased the size of myofiber, it was not statistically significant. Remarkably, oligonol significantly activated phospho‐mTOR and its downstream pathways. We also observed that the MuRF1 level was not affected by OVX, but it was significantly increased when the OVX was followed by oligonol treatment. Thus, we found that oligonol played a role in increasing the protein turnover by activating the mTOR pathway, as well as by stimulating the expression of FOXO and MuRF1 as components of the ubiquitin–proteasome pathway. These results suggested that lean mass loss after OVX without oligonol treatment did not affect the body composition because of the activation of proteolytic pathways. Previous *in* vitro and in vivo studies indicated that oligonol is beneficial in reducing muscle atrophy by attenuating proteolysis (Chang et al., 2019; Liu et al., 2017). It was assumed that these effects differ depending on the administration period and usage. Therefore, it is necessary to determine optimal oligonol regimens in further investigation.

Decreased mitochondrial function during aging is commonly involved in promoting the progression of sarcopenia (Gan et al., 2018). Consistent with a previous report (Sutham et al., 2018), we observed in this study that OVX causes changes in the mitochondrial cristae structure. However, oligonol treatment increased the number and size of mitochondria. It is commonly known that altered mitochondrial quality control processes result in mitochondrial morphological abnormalities that lead to a decreased performance and sarcopenia (Giacomello et al., 2020; Seo et al., 2010). In this study, the expression levels of two proteins involved in mitochondrial biogenesis, PGC‐1α and NRF2, were decreased after OVX; however, this effect was alleviated when OVX was followed by oligonol treatment. In mitochondrial dynamics, OPA1 and MFN2 are critical for mitochondrial inner and outer membrane fusion, and DRP1 is involved in mitochondrial fission. Other studies have shown that the progression of sarcopenia involves impaired mitochondrial quality, which is marked by the reduction or loss of OPA1, MFN2, and DRP1 expression (Favaro et al., 2019; Sebastian et al., 2016; Tezze et al., 2017). In this study, we found that the OPA1 expression level was decreased in ovariectomized rats. It has been previously shown that oligonol stimulates the mRNA levels of OPA1, MFN2, and DRP1 (Chang et al., 2019). Similarly, we found in our study that oligonol increased the expression of proteins related to mitochondrial dynamics. Thus, oligonol has the potential to improve muscle function by enhancing mitochondrial quality control processes, which prevents the loss of muscle mass and bone.

In conclusion, our results suggested that the initial state of estrogen insufficiency diminished muscle function due to decreased mitochondrial biogenesis and dynamics despite an increase in muscle mass. Furthermore, oligonol alleviated the disruption of mitochondrial biogenesis and dynamics in the skeletal muscle. We propose that oligonol improves sarcopenia progression by ameliorating dysregulation associated with sarcopenic obesity, protein turnover, and mitochondrial quality control in an estrogen‐deficiency model.

## CONFLICT OF INTEREST

The authors declare no competing interests.
